# Sugar and artificially sweetened beverage consumption and adiposity changes: National longitudinal study

**DOI:** 10.1186/s12966-015-0297-y

**Published:** 2015-10-26

**Authors:** Anthony A Laverty, Lucia Magee, Carlos A. Monteiro, Sonia Saxena, Christopher Millett

**Affiliations:** Department of Primary Care and Public Health, School of Public Health, Imperial College London, Reynolds Building, St Dunstan’s Road, London, W6 8RP United Kingdom; Departamento de Nutrição, Faculdade de Saúde Pública, Universidade de São Paulo, São Paulo, Brazil

## Abstract

**Background:**

In response to increasing policy action and public concern about the negative health effects of sugar-sweetened beverages (SSBs), there is increased promotion of artificially sweetened beverages (ASBs). These have been linked with obesity and diabetes in recent experimental work. This study examined associations between SSB and ASB consumption and changes in adiposity in a nationally representative sample of UK children.

**Methods:**

We conducted a longitudinal study of 13,170 children aged 7–11 years in the UK Millennium Cohort Study, collected in 2008 and 2012. Logistic regression was used to assess socio-demographic and behavioural correlates of weekly SSB and ASB consumption at 11 years. Linear regression examined associations between SSB/ASB consumption and changes in adiposity measures between 7 and 11 years.

**Results:**

Boys were more likely to consume SSBs weekly (62.3 % v 59.1 %) than girls at age 11 years. South Asian children were more likely to consume SSBs weekly (78.8 % v 58.4 %) but less likely to consume ASBsweekly (51.7 % v 66.3 %) than White children. Daily SSB consumption was associated with increases in percentage body fat between ages 7 and 11 (+0.57 %, 95 % confidence intervals 0.30;0.83). Daily ASB consumption was associated with increased percentage body fat at age 11 (+1.18 kg/m^2^, 0.81;1.54) and greater increases between ages 7 and 11 (+0.35 kg/m^2^, 0.09;0.61).

**Conclusion:**

Consumption of SSBs and ASBs was associated with BMI and percentage body fat increases in UK children. Obesity prevention strategies which encourage the substitution of SSBs with ASBs may not yield the adiposity benefits originally intended and this area should be a focus for further research.

## Background

Sugar sweetened beverage (SSB) consumption is an established risk factor for overweight and obesity and type 2 diabetes [1]. A meta-analysis of available longitudinal data indicates that children consuming one SSB each day are 55 % more likely to be overweight compared to those with limited consumption [1]. A cross-national study of 75 countries found that a 1 % increase in soft drink consumption is associated with an additional 4.8 cases of overweight and 2.3 cases of obesity per 100 adults [2]. Findings from a meta-analysis of prospective cohort studies found that individuals consuming 1–2 servings of SSB each day had a 26 % greater risk of developing type 2 diabetes than those with no or very low consumption [3].

Although policy responses to this evidence remains limited, restriction of marketing and promotion of SSBs to children, reducing access to SSBs at school and taxation increases have been instituted in some jurisdictions [4–6]. While these interventions appear to show promise in reducing SSB consumption, they may induce substitution to lower calorie, artificially sweetened beverage (ASB) products. ASBs are increasingly being marketed by the soft drinks industry in response to increasing scope for regulation and declining consumer preferences for SSBs in some settings [7, 8].

Positive associations between use of artificial sweeteners and markers of obesity and diabetes have been identified in recent experimental work in mice and humans [9]. A limited number of small observational studies examining associations between ASB consumption and adiposity in children have produced mixed findings and none have used nationally representative, longitudinal data [10, 11]. This study has two objectives:- (1) to document the prevalence and correlates of SSB and ASB consumption at age 11 years and; (2) examine associations of SSB/ASB consumption with adiposity at age 11 and with changes in adiposity between 7 and 11 years in a large nationally representative sample of UK children.

## Methods

### Sample and data

The Millennium Cohort Study (MCS) is a longitudinal national birth cohort study of children born in the United Kingdom (UK) between September 2000 and January 2002, alive and living in the UK at age 9 months, and eligible to receive child benefit [12]. Children were sampled using a stratified cluster sampling framework. Smaller population groups were over-sampled for the survey, including children living in disadvantaged areas, those from ethnic minority backgrounds and those living in the smaller nations of the UK. Interviews with caregivers were conducted when children were aged nine months and follow up data is currently available for four additional survey waves when children were aged 3, 5, 7 and 11 years. The survey covers a wide range of topics related to the social, economic and health circumstances of children in the UK. Full details of the study are published elsewhere [13]. Administration of questionnaires and direct measurements are carried out at home by trained interviewers using standardized protocols.

This study uses data from waves 4 and 5 of the MCS when children in the cohort are aged 7 and 11 years, collected in 2008 and 2012. The wave 5 sample consisted of 13,287 main cohort children. We excluded 117 children who were missing data for SSB and ASB consumption leaving a sample of 13,170 children. Data was analysed in 2015.

### Variables

Our main exposure variable was caregiver reporting of consumption of SSBs and ASBs at age 11 years. This was based on answers to the questions: (1) *How often, if at all, does [name] drink sweetened drinks e.g. cola, squash or Sunny Delight?* and (2) *How often, if at all, does [name] drink artificially sweetened drinks e.g. diet cola, sugar-free squash?* There were seven possible answers to the question ranging from “never” to “more than once a day”. Exposure was categorised into three groups of never/less than once a week; one to six times a week (weekly); and at least once a day (daily). This was in order to assess possible dose–response in any relationships between consumption and adiposity, and also this grouping provided adequate numbers in each group to ensure stability of estimates.

No specific questions on ASB or SSB consumption were asked in the age 7 surveys and so analyses utilising change in beverage consumption were not possible. Our continuous outcome measures were Body Mass Index (BMI) and percentage body fat objectively measured by trained interviewers. Height was measured using a Leicester stadiometer to the nearest millimetre, while weight and percentage body fat were measured using Tanita BF-522 W scales.

Other data was also based on caregiver reporting, and we attempted to control for a variety of factors which have been associated with adiposity among children in other work, including socio-demographics, dietary behaviours and physical activity [14–16]. Specifically, covariates included in fully-adjusted models were: age (in months), sex, ethnic group (White, South Asian, Black,), equivalised household income (based on the Organisation for Economic Co-operation and Development and categorised as those above and below 60 % of median income as a marker of poverty [17]), mother’s highest educational qualification (no formal qualifications, GCSE (usually completed at 16 years), A-levels (usually completed at 18 years), university or equivalent), country (England, Wales, Scotland, Northern Ireland), portions of fruit consumed per day (<3 vs. ≥3 pieces per day), breakfast consumption (seven days per week vs. not), days per week of sport/exercise (< once a week, 1–2 times a week, 3+ times a week), hours spent watching TV per weekday (<2 h vs. ≥ 2 h) and mode of transport to school (walk, cycle, public transport vs. car). No other dietary data was available from the age 11 MCS data although we additionally controlled for snacking behaviour between meals at age 7 (from none to all three of “cakes and sweet biscuits”, “crisps and other similar snacks” and “sweets or other chocolate”) and being on a controlled diet to lose weight at age 7 (answers of “*to lose or control weight*” to the question *“During the past 12 months have you controlled the type or amount [name] eats or drinks for any of these reasons).*

### Statistical analysis

Descriptive statistics were used to examine the distribution of exposure, outcomes and socio-demographic factors. Logistic regression was used to assess correlates of sweetened beverage consumption at least once a week (SSBs and ASBs considered separately) at age 11 years. Associations between categorical sweetened beverage consumption and BMI and percentage body fat at age 11 years were examined in cross-sectional analyses using linear regression. Adiposity at age 7 was then fitted in models to examine associations between sweetened beverage consumption and changes in adiposity between ages 7 and 11 years. Survey weights provided for the Millennium Cohort Study were used to correct for sampling, attrition between waves and non-response and to ensure national representativeness of anlayses [13]. These survey weights are constructed by the MCS survey team for each wave of the survey and are based on a logistic model of non-response, based on (among others) sex, mother’s age at first birth, housing tenure, ethnicity and caregiver work status [18].We performed various sensitivity analyses to assess the robustness of our results. We repeated BMI analyses using BMI-Z scores as the outcome (constructed from standard reference equations [19]). We adjusted for height and height squared in models with body fat as the outcome measure, as previous work indicates there may be a quadratic association between body fat mass and height [20, 21]. Exposure to ASBs and SSBs was not mutually exclusive in the sample as a large number of children (40.7 %) were consuming both. We therefore conducted analyses using the following four way categorization of weekly consumption (due to sample size constraints): consuming neither (16.4 % of sample); consuming ASB but not SSB at least once a week (22.8 % of sample); consuming SSB but not ASB at least once a week (20.0 % of sample); consuming both ASB and SSB at least weekly (40.8 % of sample). Finally we performed analyses excluding children who were obese (classified either by BMI-Z score as obese or in the top 5 % of the distribution of percentage body fat [22]) in order to examine possible reverse causality whereby obese children moved towards ASBs. All analyses were conducted in Stata v12.1.

As this study involved secondary analysis the MCS ethical approval was not required. Data collection for the MCS has ethical approval from the Yorkshire and Humber ethics committee and further details are available from http://www.cls.ioe.ac.uk/.

## Results

The sample of 13,170 children was 49.5 % female (Table 1) 82.9 % white, 9.6 % South Asian and 3.2 % Black. Most of the sample lived in England (64.8 %) with the fewest living in Northern Ireland (9.9 %). Three or more portions of fruit per day were eaten by 41.7 % and 86.6 % reported eating breakfast seven days per week. Partaking in sport or exercise on three or more days per week was reported by 32.1 % of the sample and 39.1 % watched TV for two or more hours every weekday.

**Table 1:** Characteristics of sample at age 11 years

		*N* = 13,170
Sex (%)	Girls	49.5
Ethnic group (%)	White	82.9
	South Asian	9.6
	African	3.2
	Missing	4.3
Equivalised income (%)	Below 60 % median	20.7
Maternal educational qualifications (%)	No formal education	15.1
	GCSE (taken at age 16)	41.4
	A-level or Diploma (taken at age 18)	24.5
	University	19.0
Country (%)	England	64.8
	Wales	14.1
	Scotland	11.2
	Northern Ireland	9.9
Fruit intake (%)	≥3 portions per day	41.7
Eats breakfast (%)	Eats breakfast 7 days a week	86.6
Diet at age 7 years (%)	On controlled diet to lose weight	5.1
Snacking between meals (crisps, sweets and chocolate, cakes and biscuits) at age 7 years (%)	None	19.6
	One	38.3
	Two	30.2
	Three	11.9
Sporting physical activity (%)	Less than once per week	25.9
	1-2 days per week	42.0
	≥3 days per week	32.1
Daily TV watching (%)	≥2 h	39.1
Mode of travel to school (%)	Walk, cycle or use bus	58.7
SSB consumption (%)	<once a week/never	39.3
	1 - 6 days a week	29.5
	At least once a day	31.2
ASB consumption (%)	<once a week/never	36.4
	1 - 6 days a week	24.2
	At least once a day	39.4
BMI (SD)	Mean	19.1 (3.4)
Weight status by BMI (%)	Normal weight	70.1
	Overweight	11.0
	Obese	18.9
% Body fat (SD)	Boys	20.1 (7.2)
	Girls	24.6 (7.1)
Change in BMI age 7 to 11 years	Mean	+2.6 (2.1)
Change in % body fat age 7 to 11 years	Mean	+1.3 (4.9)

SSB – Sugar-sweetened beverages. ASB – Artificially-sweetened beverages. BMI – Body Mass Index

SD – Standard Deviation. OECD – Organization for Economic Cooperation and Development

SSBs were consumed 1 to 6 times a week by 29.5 % of the sample and 31.2 % at least once a day. ASBs were consumed 1 to 6 times a week by 24.5 % and 39.4 % at least once a day. The mean BMI in the sample at age 11 years was 19.1 kg/m^2^ with 11.0 % of children classified as overweight and 18.9 % as obese using internationally accepted cut-points for children [23]. Mean percentage body fat was 20.1 % (standard deviation 7.2) among boys and 24.6 % (7.1) among girls.

### Correlates of weekly sweetened beverage consumption

Correlates of weekly SSB and ASB consumption are shown in Table 2. Boys were more likely to drink SSBs than girls (62.3 % vs. 59.1 %), and South Asian children were more likely to consume SSBs than White children (78.8 % vs. 58.4 %). Children in families below 60 % of the OECD median income were more likely to drink SSBs (73.8 % vs. 57.4 %) and there was indication of an inverse relationship with maternal qualifications (*e.g.* 73.9 % among mothers with no formal qualifications vs. 52.5 % among those with a university qualification). Children eating breakfast every day and those eating three or more portions of fruit per day were less likely to drink SSBs (*e.g.* 63.8 % among those eating less than three portions of fruit vs. 56.5 % among those eating three or more). Children watching two or more hours of TV on a weekday were more likely to consume SSBs (64.9 % vs. 58.1 %).

**Table 2:** Correlates of weekly sugar and artificially sweetened beverage consumption at age 11 years

		% drinking SSB	AOR	95 % CI	% drinking ASB	AOR	95 % CI
Sex	Boys	62.3	ref	ref	64.2	ref	ref
	Girls	59.1	0.86	0.79; 0.95	63.0	0.97	0.88; 1.07
Ethnic group	White	58.4	ref	ref	66.3	ref	ref
	South Asian	78.8	2.10	1.73; 2.56	51.7	0.58	0.47; 0.71
	Black	62.8	0.96	0.72; 1.28	44.0	0.42	0.29; 0.59
Income	Normal	57.4	ref	ref	63.4	ref	ref
	< 60 % OECD median	73.8	1.32	1.15; 1.5	64.5	0.99	0.86; 1.15
Mothers' level of education	No formal education	73.9	ref	ref	65.9	ref	ref
	GCSE	61.3	0.78	0.66; 0.93	70.3	1.06	0.89; 1.26
	A-level or Diploma	56.5	0.71	0.59; 0.85	62.7	0.78	0.65; 0.93
	University	52.5	0.64	0.52; 0.77	50.3	0.47	0.38; 0.57
Country	England	62.0	ref	ref	60.5	ref	ref
	Wales	58.9	1.00	0.87; 1.15	72.2	1.32	1.15; 1.52
	Scotland	57.5	0.98	0.85; 1.13	63.0	1.12	0.93; 1.34
	Northern Ireland	59.2	1.07	0.91; 1.27	68.7	1.23	1.04; 1.45
Portions of fruit eaten per day	<3	63.8	ref	ref	65.8	ref	ref
	≥ 3	56.5	0.83	0.75; 0.92	60.5	0.84	0.76; 0.93
Breakfast consumption	Eats breakfast < 7 days per week	71.2	ref	ref	70.8	ref	ref
	Eats breakfast 7 days per week	59.1	0.58	0.51; 0.67	62.5	0.87	0.75; 1.01
Days per week active	Less than once per week	64.8	ref	ref	63.8	ref	ref
	1-2 days per week	60.3	0.94	0.83; 1.06	64.8	1.00	0.88; 1.12
	≥3 days per week	58.1	1.04	0.92; 1.17	61.9	0.93	0.8; 1.07
Hours per weekday spent watching TV	<2 h	58.1	ref	ref	61.4	ref	ref
	≥2 h	64.9	1.25	1.14; 1.37	67.1	1.18	1.07; 1.31
Travel to school	By car	58.5	ref	ref	62.2	ref	ref
	By active travel	62.3	1.11	1.01; 1.22	64.7	1.14	1.04; 1.25

Weekly consumption = 1–6 days per week. SSB – Sugar-sweetened beverages, ASB – Artificially-sweetened beverages, AOR – Adjusted Odds Ratio, CI – Confidence intervals, OECD – Organization for Economic Co-operation and Development

South Asian and black children were less likely to drink ASBs than White children (51.7 %, 44.0 % vs. 66.3 % respectively). Children of mothers with a university qualification were less likely to drink ASBs than those with no formal qualifications (50.3 % vs. 65.9 %). Children eating breakfast every day and those eating three or more portions of fruit per day were less likely to drink ASBs (*e.g.* 62.5 % among those eating breakfast every day vs. 70.8 % among those not eating breakfast every day). Children watching TV for two or more hours on weekdays were more likely to drink ASBs (67.1 % vs. 61.4 %).

### Sweetened beverage consumption and adiposity

Associations between SSB consumption and adiposity are shown in Table 3. In fully adjusted longitudinal analyses weekly (+ 0.20 kg/m^2^, 0.10 to 0.31) and daily (+0.22 kg/m^2^, 0.11 to 0.34) consumption of SSBs was associated with greater increases in BMI between the ages of 7 and 11 years. Both weekly (+0.45 %, 0.21 to 0.69) and daily (+0.57 %, 0.30 to 0.83) consumption of SSBs was associated with greater increases in percentage body fat between the ages of 7 and 11 years in fully adjusted longitudinal analyses. In fully adjusted cross sectional analyses both weekly (+0.37 %, 0.05 to 0.70) and daily (+0.54 %, 0.17 to 0.92) consumption of SSBs was associated with increased percentage body fat at age 11 years.

**Table 3:** Associations between sugar-sweetened beverage (SSB) consumption and adiposity measures

		Unadjusted coefficient at age 11 (95 % CI)	Adjusted coefficient at age 11 (95 % CI)	Unadjusted coefficient age 7–11 (95 % CI)	Adjusted coefficient age 7–11 (95 % CI)
Body Mass Index					
*N*		12,368	10,443	11,318	10,283
SSB consumption^a^	Weekly	0.12 (−0.03; 0.28)	0.12 (−0.03; 0.26)	0.24 (0.14; 0.34)	0.20 (0.10; 0.31)
	Daily	0.15 (−0.03; 0.32)	0.13 (−0.04; 0.30)	0.29 (0.19; 0.40)	0.22 (0.11; 0.34)
Percentage body fat					
*N*		12,370	10,231	10,844	9851
SSB consumption^a^	Weekly	0.38 (0.03; 0.74)	0.37 (0.05; 0.70)	0.49 (0.25; 0.73)	0.45 (0.21; 0.69)
	Daily	0.52 (0.15; 0.89)	0.54 (0.17; 0.92)	0.61 (0.35; 0.86)	0.57 (0.30; 0.83)

^a^Reference group children consuming sugar sweetened beverages less than once a week/never

Weekly consumption = 1–6 days a week, Daily consumption = once a day or more than once a day

CI – Confidence intervals. Models adjusted for: age (in months), sex, ethnic group, equivalised income, mother’s highest educational qualification, country, portions of fruit consumer per day, breakfast consumption, days per week of sport/exercise, hours spent watching TV per weekday, mode of transport to school, being on a controlled diet at age 7 and snacking at age 7. Models of change in adiposity adjusted for adiposity at age 7

**Fig. 1:**
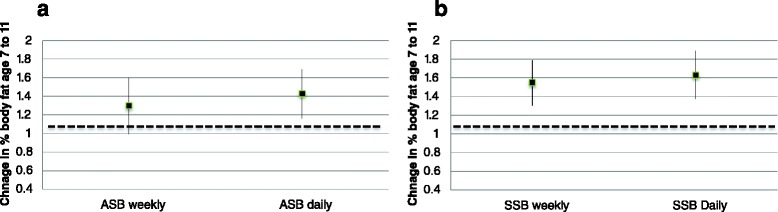
Change in percentage body fat between age 7 and 11 by ASB consumption **a** and SSB consumption (**b**). Dashed horizontal lines represent growth in percentage body fat for children drinking ASB/SSB less than once a week. * These estimates from regression model adjusted for age (in months), sex, ethnic group, equivalised income, mother’s highest educational qualification, country, portions of fruit consumer per day, breakfast consumption, days per week of sport/exercise, hours spent watching TV per weekday, mode of transport to school, snacking behavior at age 7, being on a controlled diet at age 7 and percentage body fat at age 7

Associations between ASB consumption and adiposity are shown in Table 4 and Fig. 1. In fully adjusted longitudinal analyses daily ASB consumption (+0.17 kg/m^2^, 0.06 to 0.28) was associated with greater increases in BMI between the ages of 7 and 11 years. Daily ASB consumption was associated with greater increases in percentage body fat (+0.35 %, 0.09 to 0.61) between 7 and 11 years in fully adjusted analyses. In fully adjusted cross sectional analyses both weekly (+0.47 kg/m^2^, 0.29 to 0.66) and daily (+0.58 kg/m^2^, 0.42 to 0.74) ASB consumption was associated with increased BMI at age 11 years. Weekly (+0.88 %, 0.49 to 1.27) and daily (+1.18 %, 0.81 to 1.54) ASB consumption were associated with an increased percentage body fat at 11 years.

**Table 4:** Associations between artificially sweetened beverage (ASB) consumption and adiposity measures

		Unadjusted coefficient at age 11 (95 % CI)	Adjusted coefficient at age 11 (95 % CI)	Unadjusted coefficient age 7–11 (95 % CI)	Adjusted coefficient age 7–11 (95 % CI)
Body Mass Index					
*N*		12,368	10,443	11,318	10,283
ASB consumption^a^	Weekly	0.68 (0.49; 0.87)	0.47 (0.29; 0.66)	0.16 (0.03; 0.29)	0.14 (0.00; 0.27)
	Daily	0.79 (0.64; 0.94)	0.58 (0.42; 0.74)	0.22 (0.12; 0.32)	0.17 (0.06; 0.28)
Percentage body fat					
*N*		12,370	10,231	10,844	9851
ASB consumption^a^	Weekly	1.29 (0.89; 1.70)	0.88 (0.49; 1.27)	0.32 (0.01; 0.63)	0.26 (−0.04; 0.55)
	Daily	1.48 (1.12; 1.83)	1.18 (0.81; 1.54)	0.39 (0.15; 0.63)	0.35 (0.09; 0.61)

^a^Reference group children consuming artificially sweetened beverages less than once a week/never

Weekly consumption = 1–6 days a week, Daily consumption = once a day or more than once a day

CI – Confidence intervals. Adjusted models adjusted for: age (in months), sex, ethnic group, equivalised income, mother’s highest educational qualification, country, portions of fruit consumer per day, breakfast consumption, days per week of sport/exercise, hours spent watching TV per weekday, mode of transport to school, being on a controlled diet at age 7 and snacking at age 7. Models of change in adiposity adjusted for adiposity at age 7

### Sensitivity analyses

Analyses using BMI Z-scores as our outcome measure produced similar results (Appendix Table 5). Analyses of percentage body fat adjusting for both height and height squared were also similar to main analyses (Appendix Table 6). Analyses using an exposure variable with separate categories for weekly ASB and SSB consumption produced findings which were consistent with those from our main analyses (Appendix Table 7). Analyses after excluding children who were obese at age 11 (Appendix Table 8) found attenuated associations between artificially sweetened beverage consumption and adiposity, although these remained statistically significant.

## Discussion

This study used data from the UK Millennium Cohort Study to examine associations between SSB and ASB consumption and adiposity changes in UK children between 7 and 11 years. This age period is important as it is associated with accelerated weight gain [24]. We found that regular consumption of SSBs and ASBs was associated with greater increases in BMI and percentage body fat. These results remained statistically significant after adjusting for a variety of socio-demographic and behavioral factors associated with an increased risk of adiposity. ASB and SSB consumption was lower among children of mothers with higher educational qualifications and SSB consumption was higher among south Asian children. Children who did not eat breakfast every day and those watching more than two hours of TV per day were more likely to drink both ASBs and SSBs.

While SSB consumption is an established risk factor for adiposity [1], few studies have examined associations with ASBs. A regional study of 1200 children in England found a positive association between ASB consumption and changes in adiposity between 5 and 9 years [25]. However, the authors suggest that this finding may reflect reverse causality due to overweight children switching to ASBs as part of an unsuccessful weight loss strategy. Associations between ASB consumption and adiposity remained statistically significant in our sensitivity analysis which excluded children who were obese at age 11 years. This suggests that our findings are not fully explained by substitution to ASBs among heavier children. Our findings are also consistent with those from an intervention trial which found that substitution of SSBs with water resulted in substantially lower calorie intake when compared with substitution with ASBs [26] while an intervention directed at adolescents encouraging substitution to ASBs was not associated with lower BMI than controls [27]. These latter findings support the conclusion that in the absence of energy restrictions, switching to ASBs is not an effective strategy for weight loss [28].

A variety of possible mechanisms by which artificial sweeteners may be linked to weight gain have been proposed: such sweeteners may increase appetite and lead to increased consumption, or they may train the palate to enjoy similar sweet foods, which may or may not be low calorie [28, 29]. Another possible explanation is that people may consciously overcompensate for these low calorie options and overindulge in other intakes [28, 29]. There is at present no conclusive evidence on these possible mechanisms, and the area remains an open area of research [10, 28]. In addition to adiposity, ASBs have also been linked to other health outcomes, such as incident diabetes in a recent meta-analysis [30].

This study is the first national longitudinal examination of associations between SSBs and ASBs and adiposity among pre-adolescent children. We used two objective measures of adiposity in children and found similar associations for both which strengthens the findings. The MCS is designed to be representative of the population of the UK and the percentage of our sample which was overweight or obese is similar to officially published estimates. 29.9 % of our sample was overweight or obese, which is similar to data from the National Child Measurement Programme for England which found 33.3 % of age 10 and 11 year old children to be overweight or obese in 2012/13 [31]. Nonetheless, there are a number of study limitations that should be considered. Measurement of beverage consumption was based on caregiver reporting, as were potential confounding variables. The MCS is not specifically designed with a nutrition focus and it remains possible that there are other important elements of diet which we have not been able to control for. In particular, inclusion of data on overall diet quality would have been useful in exploration of these issues. However, there was no data available on overall diet quality, and the data we use on snacking behavior comes from age 7 and may not accurately reflect snacking at age 11.This means that residual confounding remains a possible explanation for these findings, especially given that adjustment reduced effect sizes. Nonetheless we did control for a wide range of factors related to adiposity in previous work.Our predictor variable was based on sweetened beverage consumption at age 11 years, rather than at both time points in the study. Inclusion of data on consumption from both time points would have allowed less cautious interpretation of the results presented here and without this further research is needed to determine potential associations between these beverages and adiposity in children. Although parents were asked about beverage consumption at age 7 years, the question referred only to intake between meals and did not discriminate between artificial and sugar sweetened beverages. We fitted a categorical variable for exposure to SSBs or ASBs which meant that there was some sweetened beverage consumption in the reference group (at most once a month). Exposure to ASBs and SSBs was not mutually exclusive in the sample as a large number of children (40.7 %) were consuming both. However, findings from sensitivity analysis which involved fitting a four way categorization where weekly SSB/ASB consumption was mutually exclusive were consistent with our main findings. Although changes in adiposity seen were modest and may be accounted for by measurement error at the individual level, given our sample size it is unlikely that our findings areentirely due to artefact. Our findings may be partly explained by reverse causality *e.g.* more adipose children switching to ASBs as part of an unsuccessful weight loss strategy. However, associations between ASB and adiposity were statistically significant in sensitivity analyses which excluded obese children.

The World Health Organization recently published draft guidance on sugar intake; that adults should keep their intake below 10 % of total calories. The guidance also states that reducing sugar intake to below 5 % of total calories would yield additional population health benefits [32]. The WHO guidance is underpinned by robust epidemiological evidence indicating sugar consumption as an important cause of excess weight gain in both adults and children globally [1, 33]. Reducing SSB consumption offers considerable scope to achieve the WHO recommendations as SSBs account for up to 10 % of children’s energy intake in the UK [25]. Policy options to reduce intakes include upstream interventions such as cap and trade policies on adding sugar to the food chain, similar to the international carbon trading system [34]. Other proposed interventions include warning labels on SSBs [35], removing such drinks from schools [36] or additional taxes on such drinks (recently passed in Berkeley California). Findings from this and previous research indicate that encouraging substitution of SSBs with ASBs may not yield the health benefits originally intended in terms of adiposity or diabetes and substitution with water may be preferable Although evidence on policy impacts is emerging, the ubiquity of both SSBs and ASBs suggests that a variety of interventions are likely to be required in tandem, and that national governments will need support, co-ordination and guidance from international organizations [37]. Additionally, synergies identified between behaviors such as screen viewing and consumption [38], as well as the importance of physical activity [21] suggest that multi-faceted interventions tackling a variety of risk factors are likely to be be needed. Given our findings that consumption of sweetened beverages is higher in lower socio-economic and ethnic minority groups, it is important that any impacts on inequalities in childhood obesity are carefully monitored.

## Conclusion

This national longitudinal study found that both sugar and artificially sweetened beverage consumption was associated with increases in adiposity in pre-adolescent children. These findings strengthen a limited evidence base suggesting that artificially sweetened drinks may be contributing to, rather than constraining population level increases in adiposity. Obesity prevention strategies which aim to encourage substitution of SSBs with ASBs may not yield the benefits intended and this should be a focus for further research.

## Appendix

**Table 5:** Associations between sweetened beverage consumption and BMI Z-score

		Unadjusted coefficient at age 11 (95 % CI)	Adjusted coefficient at age 11 (95 % CI)	Unadjusted coefficient age 7–11 (95 % CI)	Adjusted coefficient age 7–11 (95 % CI)
Sugar sweetened beverages					
Sugar sweetened beverage consumption at age 11^a^	At least once a week	0.04 (0.00; 0.10)	0.04 (0.00; 0.09)	0.08 (0.04; 0.12)	0.07 (0.02; 0.11)
	At least daily	0.05 (0.00; 0.11)	0.04 (−0.02; 010)	0.11 (0.07; 0.14)	0.08 (0.04; 0.12)
Artificially sweetened beverages					
Artificially sweetened beverage consumption at age 11^b^	At least once a week	0.23 (0.16; 0.29)	0.17 (0.10; 0.24)	0.05 (0.01; 0.10)	0.04 (−0.01; 0.10)
	At least daily	0.25 (0.19; 0.30)	0.19 (0.12; 0.25)	0.05 (0.01; 0.09)	0.04 (0.01; 0.08)

^a^Reference group children consuming sugar sweetened beverages less than once a week/never

^b^Reference group children consuming artificially sweetened beverages less than once a week/never

Weekly consumption = 1–6 days a week, Daily consumption = once a day/ more than once a day

CI – Confidence intervals. Adjusted models adjusted for: ethnic group, equivalised income, mother’s highest educational qualification, country, portions of fruit consumer per day, breakfast consumption, days per week of sport/exercise, hours spent watching TV per weekday and mode of transport to school, being on a controlled diet at age 7, snacking at age 7, and height squared. Models of change in adiposity adjusted for adiposity at age 7

**Table 6:** Associations between sweetened beverage consumption and percentage body fat, analyses including adjustment for height and height squared

		Unadjusted coefficient at age 11 (95 % CI)	Adjusted coefficient at age 11 (95 % CI)	Unadjusted coefficient age 7–11 (95 % CI)	Adjusted coefficient age 7–11 (95 % CI)
Sugar sweetened beverages					
Sugar sweetened beverage consumption at age 11^a^	Weekly	0.34 (0.00; 0.68)	0.31 (−0.01; 0.64)	0.48 (0.24; 0.73)	0.42 (0.18; 0.67)
	Daily	0.57 (0.20; 0.94)	0.52 (0.15; 0.88)	0.61 (0.35; 0.86)	0.54 (0.28; 0.80)
Artificially sweetened beverages					
Artificially sweetened beverage consumption at age 11^b^	Weekly	1.20 (0.80; 1.61)	0.78 (0.39; 1.17)	0.36 (0.05; 0.67)	0.26 (−0.03; 0.56)
	Daily	1.39 (1.03; 1.74)	1.04 (0.69; 1.39)	0.40 (0.16; 0.64)	0.33 (0.08; 0.59)

^a^Reference group children consuming sugar sweetened beverages less than once a week/never

^b^Reference group children consuming artificially sweetened beverages less than once a week/never

Weekly consumption = 1–6 days a week, Daily consumption = once a day/more than once a day

CI – Confidence intervals. Adjusted models adjusted for: age (in months), sex, ethnic group, equivalised income, mother’s highest educational qualification, country, portions of fruit consumer per day, breakfast consumption, days per week of sport/exercise, hours spent watching TV per weekday and mode of transport to school, being on a controlled diet at age7and snacking at age 7. Models of change in adiposity adjusted for adiposity at age 7

**Table 7:** Associations between weekly sugar-sweetened beverage consumption and adiposity measures

	Unadjusted coefficient for adiposity at age 11 (95 % CI)	Adjusted coefficient for adiposity at age 11 (95 % CI)	Unadjusted coefficient for change in adiposity between 7 and 11 (95 % CI)	Adjusted coefficient for change in adiposity between 7 and 11 (95 % CI)
Category (% of sample)	Body Mass Index			
Neither (16.4 %)	ref	ref	ref	ref
ASB but not SSB (22.8 %)	0.91 (0.70; 1.12)	0.72 (0.50; 0.95)	0.20 (0.06; 0.34)	0.13 (−0.01; 0.26)
SSB but not ASB (20.0 %)	0.24 (0.02; 0.46)	0.17 (−0.05; 0.39)	0.28 (0.13; 0.42)	0.20 (0.06; 0.34)
Both (40.8 %)	0.86 (0.69; 1.04)	0.65 (0.45; 0.86)	0.44 (0.31; 0.56)	0.33 (0.19; 0.47)
Category (% of sample)	Percentage body fat			
Neither (16.8 %)	ref	ref	ref	ref
ASB but not SSB (23.5 %)	1.60 (1.10; 2.11)	1.39 (0.86; 1.92)	0.25 (−0.10; 0.61)	0.22 (−0.13; 0.60)
SSB but not ASB (19.0 %)	0.57 (0.07; 1.08)	0.54 (0.05; 1.05)	0.46 (0.10; 0.82)	0.47 (0.10; 0.84)
Both (40.7 %)	1.78 (1.38; 2.19)	1.49 (1.05; 1.94)	0.82 (0.51; 1.12)	0.73 (0.41; 1.05)

Weekly consumption = at least one day per week

ASB – artificially sweetened beverages, SSB – sugar sweetened beverages, CI – Confidence intervals

Adjusted models adjusted for: age (in months), sex, ethnic group, equivalised income, mother’s highest educational qualification, country, portions of fruit consumer per day, breakfast consumption, days per week of sport/exercise, hours spent watching TV per weekday and mode of transport to school, being on a controlled diet at age 7 and snacking at age 7. Models of change in adiposity adjusted for adiposity at age 7

**Table 8:** Associations between sweetened beverage consumption and adiposity measures, excluding children obese at age 11

		Unadjusted coefficient at age 11 (95 % CI)	Adjusted coefficient at age 11 (95 % CI)	Unadjusted coefficient age 7–11 (95 % CI)	Adjusted coefficient age 7–11 (95 % CI)
Body Mass Index					
Artificially sweetened beverage consumption at age 11^a^	Weekly	0.28 (0.15; 0.40)	0.32 (0.18; 0.45)	0.12 (0.02; 0.22)	0.12; 0.01; 0.22)
	Daily	0.30 (0.19; 0.42)	0.31 (0.18; 0.44)	0.13 (0.04; 0.22)	0.13 (0.03; 0.23)
Percentage body fat					
Artificially sweetened beverage consumption at age 11^a^	Weekly	0.43 (0.05; 0.28)	0.47 (0.38; 0.62)	0.17 (0.05; 0.28)	0.18 (0.05; 0.30)
	Daily	0.38 (0.26; 0.50)	0.36 (0.23; 0.50)	0.16 (0.07; 0.26)	0.15 (0.06; 0.25)

^a^Reference group children consuming artificially sweetened beverages less than once a week/never

Weekly consumption = 1–6 days a week, Daily consumption = once a day/ more than once a day

Adjusted models adjusted for: age (in months), sex, ethnic group, equivalised income, mother’s highest educational qualification, country, portions of fruit consumer per day, breakfast consumption, days per week of sport/exercise, hours spent watching TV per weekday and mode of transport to school, being on a controlled diet at age 7 and snacking at age 7. Models of change in adiposity adjusted for adiposity at age 7
